# Advancing laparoscopy in resource-limited settings

**DOI:** 10.1186/s12893-024-02387-2

**Published:** 2024-03-26

**Authors:** Surafel Mulatu Djote, Daniel Ahmed Muhie, Getachew Desta Alemayehu

**Affiliations:** 1 Department of Surgery, Yekatit 12 Hospital Medical College, Addis Ababa, Ethiopia; 2 Asan Medical Center Division of Colorectal Surgery, Seoul, South Korea

**Keywords:** Laparoscopic cholecystectomy, Open cholecystectomy, Critical view, Conversion rate

## Abstract

**Background:**

Although laparoscopic surgery has made remarkable progress and become the standard approach for various surgical procedures worldwide over the past 30 years, its establishment in low-resource settings, particularly in public hospitals, has been challenging. The lack of equipment and trained expertise has hindered its widespread adoption in these settings. Cholecystectomy is one of the most commonly performed procedures using laparoscopy world wide

**Aim:**

The aim of the study is to determine whether laparoscopic cholecystectomy is feasible in a resource challenged setting

**Methods:**

The research focused on individuals who underwent laparoscopic or open cholecystectomies at Yekatit 12 Hospital in Addis Ababa, Ethiopia, over a one-year period. Comprehensive data collection was conducted prospectively, encompassing both intraoperative and postoperative parameters. Follow-up was carried out via phone calls. The surgical procedures employed innovative techniques, including the reuse of sterilized single-use equipment and the utilization of local resources. The evaluation involved a comparison of demographic information, intraoperative details (such as critical view determination and operative duration), and postoperative complications, including assessments of pain and wound infections

**Results:**

From August 2021 to September 2022, 119 patients were assessed. Among these patients, 65 (54.6%) underwent open cholecystectomies, while the remaining 54 (45.4%) underwent laparoscopic cholecystectomies. The average duration of the laparoscopic cholecystectomies was 90.7 min, which is 17.7 min behind the open. Patients in the laparoscopy group had significantly shorter hospital stays than the open group, and 94% were discharged by post operative day 2. The conversion rate from laparoscopic to open surgery was determined to be 3.3%

**Conclusion:**

To sum up, the safe execution of laparoscopic cholecystectomies is feasible in public hospitals and settings with limited resources, given adequate training and resource distribution. The study findings showcased superior outcomes, including reduced hospitalization duration and fewer complications, while maintaining comparable levels of operative duration and patient satisfaction in both groups

**Supplementary Information:**

The online version contains supplementary material available at 10.1186/s12893-024-02387-2.

## Introduction

Gallstone disease is a commonly encountered medical condition that frequently necessitates surgical treatment. In Ethiopia, there is a limited amount of data regarding the epidemiology of gallstone disease. Although studies have indicated a lower prevalence in the broader sub-Saharan region, approximately 5% compared to global rates, the increasing trends of modernization, obesity, and urbanization contribute to the surge in cholecystectomy procedures. Consequently, cholecystectomy has become the most common elective surgery in our hospital. Cholecystectomy, the surgical removal of the gallbladder, has undergone significant advancements, with laparoscopic cholecystectomy emerging as the standard approach due to its manifold advantages over open surgery.

Laparoscopic cholecystectomy is now the established standard of care for treating gallstone disease, a conclusion drawn from consistent research findings since its introduction in the early nineties. The evident superiority of this approach is attributed to a substantial decrease in the “trauma of access,” leading to a notable reduction in stress cytokines [1]. This reduction is directly associated with accelerated patient recovery and a quicker return to normal functioning. Additionally, the procedure results in a significant decrease in surgical wound size, thereby minimizing complications related to wounds, such as infections, pain, and incisional hernias [2–4]. No comparative study has been conducted in the Sub-Saharan region comparing laparoscopic versus open cholecystectomies. However, the well-established advantages of laparoscopic surgeries, generally recognized for their benefits, would be particularly advantageous in a region where the prevention of wound infections and the economic significance of swift workforce recovery are highly valued.

Nevertheless, these benefits do not come without associated risks. The laparoscopic procedure necessitates a properly functioning laparoscopy device, a consistent electricity supply, and a skilled workforce. Additionally, there is an elevated risk of injuring the common bile duct compared to the open counterpart, especially during the initial phases of its introduction [5]. 

Despite these advancements, open cholecystectomy remains the predominant surgical procedure in typical public hospital settings in Ethiopia [6]. However, the broader implementation of laparoscopic cholecystectomy in resource-limited settings like Ethiopia has been hampered by challenges arising from inadequate equipment, resources, and trained expertise [7]. 

Due to healthcare resources constraint, surgical treatment options in Ethiopia have historically faced challenges in meeting the growing demand for safe and effective procedures [8]. Despite this, there have been concerted efforts to introduce and establish laparoscopic cholecystectomy as a viable surgical option in public hospitals, especially the American College of Surgeons training Hub in Hawassa is worth mentioning [9]. In Ethiopia, few public institutions have made efforts to incorporate laparoscopy into their surgical procedures, although it has not yet become a standard service [10, 11]. In general, the implementation of laparoscopic procedures in resource-limited settings presents unique opportunities and challenges, as it requires adaptation to the local context and overcoming various barriers.

The preference for laparoscopy over open surgery has become widely accepted in numerous procedures, even in the early stages of its introduction. This preference is supported by research conducted in Western settings during its introduction in the early nineties [2, 12]. However, it is important to note that this evidence may not necessarily apply to different settings with resource limitations and unique characteristics, such as challenges related to the availability of expert training and infrastructure in the developing world. This paper aims to compare the outcome of laparoscopic cholecystectomy in relation to open cholecystectomy with predefined variables of procedure duration, post operative pain and hospital stay .

## Methods

### Study settings

The research took place at Yekatit 12 Hospital Medical College, an age-old public hospital situated in the heart of Addis Ababa, the capital of Ethiopia. This hospital is currently serving a catchment population of five million, and was giving service for a century. There are two trained laparoscopic surgeons (1 general and 1 hepatobiliary surgeons), and12 general surgeons. As OR time is shared among the surgeons there were both laparoscopic and open cholecystectomies being done at the same time by whoever is assigned for the day.

In this study modifications were used to circumvent the challenges of unavailable supplies and consumables which were extracorporeal suturing and use of disposable trocars and reusing them with adequate processing. Training for the scrub nurses was also undertaken. The camera assistants were randomly assigned residents, who were all being exposed to the procedure for the first time.

The open cholecystectomy was performed via standard right subcostal incision and for the laparoscopy group standard four port cholecystectomy was used. Surgical gloves were used in retrieving the gallbladder via epigastric port. All 10 mm ports had fascial closure with 2 − 0 vicryl. As the service started there were no available laparoscopic clips in the local market, initial procedures were performed with an extra-corporeal suture ligation of the cystic duct and artery. This step was later replaced with a proper laparoscopic clip.

### Study population

All patients who have under-went cholecystectomy at Yekatit 12 Hospital Medical College between August 2021 and September 2022.

#### Inclusion criteria

All patients that have undergone elective cholecystectomy at Yekatit 12 Hospital Medical College from August 2021 to September 2022.

#### Exclusion criteria

Patients who were treated operatively as an emergency and patients not willing to participate in the study were excluded from the study.

### Operational definition

#### Procedure duration

The total duration of the surgical procedure as recorded by the anesthesia team in minutes.

#### Post operative pain

The pain score reported by the patient measured on the morning of the first post op day, on a scale of 1–10.

#### Hospital stay

The duration of hours patient spent in the hospital measures in hours from the time of surgery to discharge.

#### Post operative complication

Acute post operative acute complication: -any complication related to the surgical intervention within the first two post operative weeks.

Chronic post operative complications: - any complications related to the surgical intervention after the second post operative week.

#### Patient satisfaction

Patient satisfaction was assessed using a Likert scale ranging from 1 to 5 during telephone interviews conducted, on average, on the 70th postoperative day. The scale ranged from 1 (very satisfied) to 5 (very dissatisfied).

### Data collection methods

Information regarding patient demographics and their initial clinical diagnoses was collected from electronic medical records. Intraoperative timing, the evaluation of critical safety views, and first post operative day pain scores were prospectively documented in individual patient electronic records by residents. Postoperative follow-up data were gathered through telephone interviews, with an average follow-up period of 69.2 days after the surgical procedures by assigned personnel.

### Data analysis methods

After completeness of the data was crosschecked using statistical software package, it entered into Epi-Data software and exported to SPSS version 26-statistical software package for further analysis.

Tables, graphs, and texts were to summarize descriptive statistics of the study. We evaluated the correlation between postoperative pain scores, the duration of hospital stay after surgery, and patient satisfaction in the context of comparing laparoscopic cholecystectomy with open cholecystectomy. All variables with a *p* < 0.25 in the bivariate analysis are entering in the logistic regression model. Odds ratios (OR) and 95% confidence intervals was then calculated a *p*-value of less than 0.05 was considered significant.

## Results

### Variable summary

There was a total of 119 patients included in the study, with 65 (54.6%) in the open group and 54 (45.4%) in the laparoscopy group with a male to female ratio of 1:4 with a mean age of 37.6 yrs. Four patients had an initial laparoscopic approach converted to open due to failure to achieve critical view of safety, and followed as open cholecystectomy. The comparison of the study groups exhibits similar characteristics in terms of their demographic and clinical parameters (Table 1).

**Table 1:** Independent variables

Age Range	Count n/%		*P-*Value
	Open	Lap	0.345
> 10		1(1.9%)	
11–25	8(12.3%)	7(13.0%)	
26–45	43(66.2%)	39(72.2%)	
46–65	12(18.5%)	4(7.4%)	
66<	2(3.1%)	3(5.6%)	
Diagnosis			0.543
Symptomatic cholelithiasis	61(93.8%)	52(96.3%)	
Others	4(6.2%)	2(3.7%)	
Operating Surgeon			0.618
General Surgeon†	63(96.9%)	52(96.3%)	
Hepatobiliary Surgeon	2(3.1%)	2(3.7%)	

† (The open cholecystectomies were done by 12 general surgeons and the laparoscopic ones were done by 1 general surgeon and hepatobiliary surgeon)

### Operative duration

The open cholecystectomy group had a mean operative time of 73.12 min, whereas the laparoscopy group had a mean operative time of 90.78 min, statistically significant with a *p* value of 0.007. Table 2.

**Table 2:** Out-come variable summary table

	Count n/%		*P*-Value
	Open	Lap	
Procedure Duration (minutes)			0.007
< 60	29(44.6%)	10(18.5%)	
61–120	34(52.3%)	39(72.2%)	
> 120	2(3.1%)	5(9.3%)	
Post Operative Pain			0.147
Mild	5(7.7%)	8(20.5%)	
Moderate	39(60.0%)	24(61.5%)	
Severe	21(5.3%)	7(17.9%)	
Post OP complications			
Acute	6(9.3%)	2(4.8%)	0.663
Chronic	8(12.3%)	4(9.8%)	0.361
Hospital Stay (Hours)			< 0.001
24	-	14(25.9%)	
25–48	-	37(68.5%)	
> 48	65(100%)	3 (5.6%)	

### Critical view of safety

As a beginner of laparoscopic procedures, attention was given to achieving critical view of safety in all patients and 47 patients of the 54 laparoscopic group (87%) had critical view of safety achieved and there were no bile duct injuries encountered.

### Post operative pain score

The laparoscopy group had generally a lower pain scores (average of 4.9/10) registered in the immediate post operative day than open group 5.6/10 recorded on the first post operative day.

### Post operative acute and chronic complications

Both acute and chronic complications were observed in both groups.

In terms of acute postoperative complications, among those interviewed in the open surgery group, 4 patients (6%) experienced wound infection, while in the laparoscopy group, 1 patient (2.4%) had a trocar site infection at umbilical port site. Additionally, 1 patient (1.5%) in the open surgery group developed swelling around the wound, whereas no other acute complications were reported in the laparoscopy group.

Turning to chronic postoperative complications, the most common issue was pain. In the open surgery group, 4 patients (6.2%) reported experiencing episodes of pain, while in the laparoscopy group, 4 patients (9.8%) reported the presence of pain. Furthermore, there were 2 patients (3.1%) in the open surgery group who complained of swelling at the surgical site. Table 2.

### Duration of hospital stay

There were an overall 233 hospital days for the open group with an average hospital day of 3.6, while the laparoscopy group had a total of 97 hospital days with an average of 1.8 hospital days, and *p* value of < 0.01. Table 2.

## Discussion

Due to its limited implementation laparoscopic surgery feasibility and outcome studies are scarce in the Ethiopian and in the wider low resource setting region [13]. This study evaluated the practicality of laparoscopic cholecystectomy in a public hospital by comparing its outcome with the more routine counter-part i.e., open cholecystectomy. Both groups were having similar patient population with comparable demographic and clinical characteristics being done in the same setting over a course of a year. Table 1.

The majority of the procedures were done by general surgeons (Open-96.9%, Laparoscopic-96.3%), the rest were done by hepatobiliary surgeon. The surgeon who had performed the laparoscopic procedures received training during his surgical residency, there were no additional trainings. This is comparable with the findings of studies that reported on their initial reports of laparoscopic service establishment, in which all the procedures were performed by a general surgeon in a private hospital setting in Ethiopia [14], and hospitals in the US [15].

One of the prominent challenges of laparoscopic services in the resource challenged setting is the issue of infrastructure and sustainable supply of consumables [11]. Local improvisations and solutions need to be sought beforehand in order to achieve a consistent service, as it has been the case in our study. However, political engagement at both the Ministry of Health and hospital administration levels is clearly essential to spearhead a nationwide initiative to provide basic laparoscopic consumables, ensuring uninterrupted and sustained service to the public.

The laparoscopic group had an operative time of 90.77 ± 26.74 min compared to 73.1 ± 24.26 min for the open group. The longer operative time in laparoscopy is attributed to instrument setup and team unfamiliarity. However, this prolonged duration correlates with quicker recovery and discharge during follow-ups. A study in Taiwan in 1993 showed similar prolonged operative times for laparoscopy compared to open cholecystectomies [2]. Our average operative time of 85.7 min is comparable to other newly established programs [15–17]. Studies in the US [15] and Uganda [16] reported mean operative times of 77 min and 40.5 min, respectively, though the latter evaluated only 10 patients. These studies involved general surgeons as operating surgeons, similar to ours, differing mainly in prior training duration and level.

**Fig. 1:**
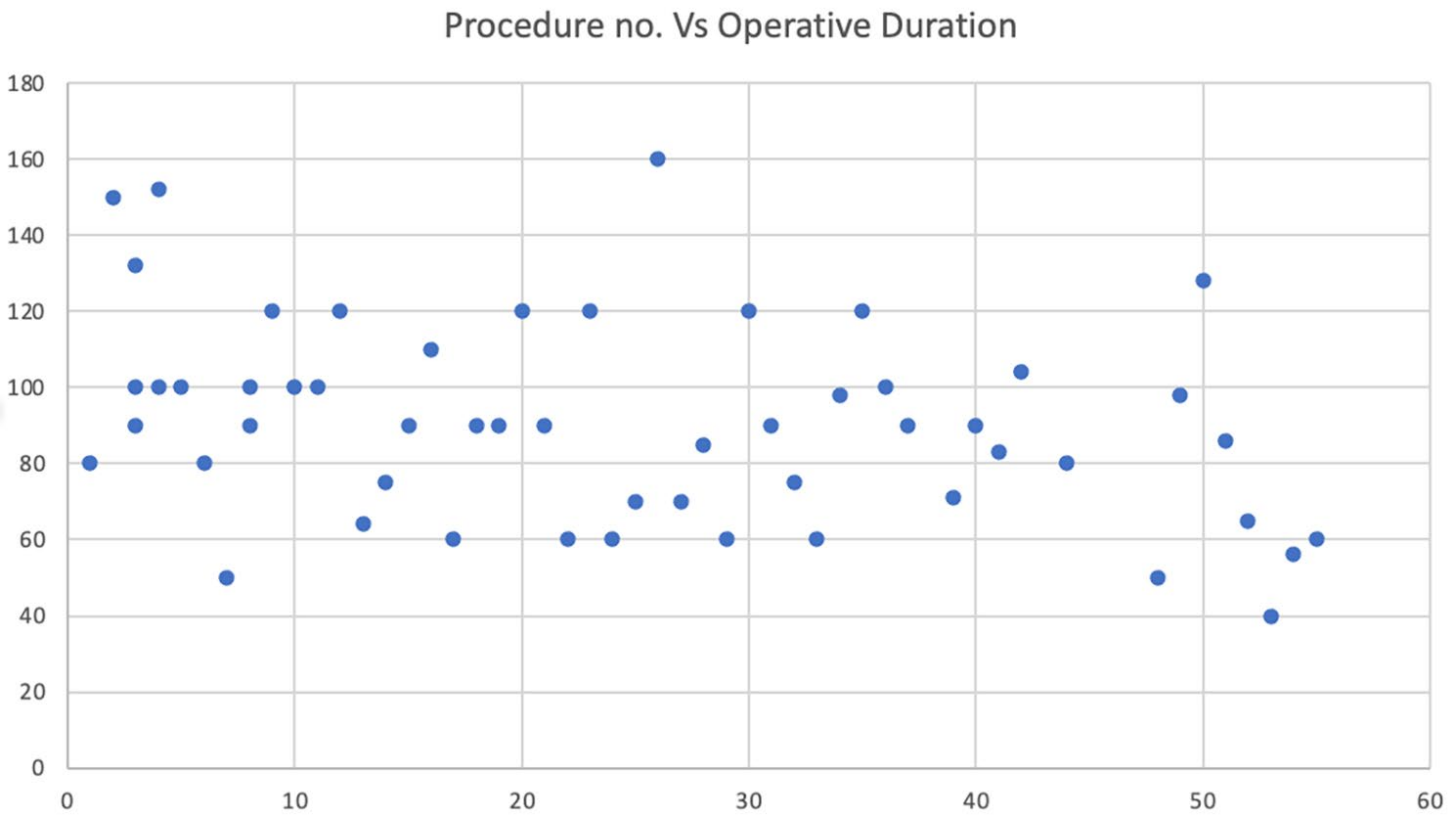
Procedure duration trend over the study periods

It is also worth noting that as the duration of the service continued the learning curve has naturally improved and hence the operative time too as depicted by the Fig. 1. This proves the point that skills are improved in a locally contextual manner in improving learning curve pattern [18]. We had a conversion rate of 3.4% which is lower than reported average which lies between 5 and 10%, this might change as procedure number keeps growing [19]. There were no bile duct nor trocar injuries encountered during the study period where studies have showed the expected rate of injury to be up to 5% initially but through the gradual development of standardized performance steps and achievement of critical view of safety, it is believed to be lowered below 1% [20].

Given that a majority of the study participants come from economically disadvantaged backgrounds, and a relatively young and economically active segment of the population with mean age of 37.6years, the reduced duration of hospitalization emerges as a noteworthy factor. The financial benefit of a shorter hospital stay post-surgery is especially conspicuous in this demographic, as they heavily depend on their daily earnings to support their families. Additionally, our study unequivocally demonstrates the advantages of the laparoscopic approach in this regard. Table 1.

Postoperative pain reduction is a key benefit of laparoscopy over open surgery, as seen in our study’s average pain score of 4.9 vs. 5.6 out of 10 [21]. While pain scoring reliability may vary due to its subjective nature, it remains an objective assessment tool [22]. Previous research comparing laparoscopic cholecystectomy to open surgery found superior postoperative pain management with laparoscopy [23], consistent with many international studies [24], despite variations in pain assessment methods.

For the follow up complications reported on the average of 70th post op day, there were less wound related complications in the laparoscopy group, especially numbness and scar were absent as expected. The port site infection rate in the laparoscopy group were 2.4%, and 6% for the open group which is up by 2.5 times.

In a prospective study in India, the reported port site infection rates for general laparoscopic procedures were 6.4%, with the umbilical port being most affected [25]. Another Indian study focusing on post-laparoscopic cholecystectomy found a 4.5% infection rate, mainly at the epigastric port [26]. While our findings align with these, there are differences in prophylactic antibiotic usage. Both studies, like ours, repurposed single-use items, suggesting that improving access to affordable surgical supplies could further reduce infection rates.

Moreover, conducting follow-up at a later date allows for the identification of additional complication risks, such as incisional hernia, which have previously been reported to be positively impacted by the laparoscopic approach [27]. 

The laparoscopy group demonstrated a significantly higher achievement of a critical view of safety (*p* = 0.021). This was done by the confirmation of the operating surgeon that cystic structures were clearly identified and dissected, as dictated by the standard i.e., dissecting the cystic plate, lifting the posterior surface of the gall bladder of the liver bed and assuring the presence of only two structures entering the gall bladder before any further steps [28]. 

This critical step ensures the establishment of precise anatomical landmarks before any cuts are made during laparoscopic cholecystectomy [29]. The critical view of safety was successfully attained in 87% of the laparoscopic cholecystectomy cases.

There were patient satisfaction rates collected from the patients on Likert’s scale which showed similar distribution that leaned toward the satisfied side of the scale. We have also looked at the responses of patients on recommendation of the procedure with a simple question and found that both groups have got positive recommendations with no statistically significant difference. Table 3.

**Table 3:** Patient satisfaction rate

		Patient Satisfaction Rate			Total
		Very satisfied	Satisfied	Neutral	
procedure type	Open	56(86.2%)	8(12.3%)	1(1.5%)	65
	Lap	33(80.5%)	5(12.2%)	3(7.3%)	41
Total		89	13	4	106

Another aspect to take into account in this study is that, given the extended waiting lists for surgeries, patients may be content with having their procedures performed through any approach and may not fully grasp the distinctions between them. It would take detailed qualitative research to assess the satisfaction rates in depth.

A similar study of patients’ satisfaction rating of laparoscopic procedures done in Cameroon has reported that patients were globally satisfied with the process of care but financial and geographical barriers should be addressed [30]. 

As depicted in Table 1a noteworthy finding in this research is the statistically significant reduction in hospital stay observed in the laparoscopic group, an average stay of 1.8 days compared to 3.6 for the open group, with a quarter of the patients discharged within 24 h and 94.4% of patients by the second postoperative day. The *p*-value is < 0.05, which adds weight to the argument for expanding the use of minimally invasive procedures in the country’s surgical practice. The US land mark study that evaluated experiences of 38 surgeons in their initial laparoscopic cholecystectomy [15] reported an average hospital stay of 1.2 days and a third of the patients discharged within less than 24 h of the study. While the Ugandan paper reported an average hospital stay of 4.2 days for the laparoscopy group.

Despite being a well-known fact, this research contributes to addressing the comparative advantage in a resource-constrained setting, which has been largely unexplored in the literature. As experience and momentum in this field continue to grow in the region, it is anticipated that future studies with larger patient populations will further substantiate these findings, a limitation of this paper.

## Conclusion

In summary, based on the findings of this research, it is evident that there are compelling reasons to promote the expansion of laparoscopic services in resource-constrained environments. Laparoscopic cholecystectomy offers distinct advantages over open cholecystectomy, particularly in terms of reduced postoperative pain and shorter hospitalization. Moreover, these results suggest that general surgeons in the region can be trained to perform the procedure with favorable patient outcomes. It is crucial to prioritize the critical aspects of procuring essential supplies and equipment while carefully allocating resources. This guarantees that the established advantages of this surgical method are easily available to the public. A plan is already in place to expand laparoscopic services at Yekatit 12 Hospital, transitioning from predominantly open cholecystectomy to primarily laparoscopic cholecystectomy and other laparoscopic procedures. This includes the ongoing training of staff surgeons and its integration into the surgery training curriculum. While acknowledging these results, it is crucial to stress the importance of conducting further studies in similar settings with larger patient populations.

### Weaknesses and strengths of the study

Acknowledging certain weaknesses, the study’s small sample size limits the strength of conclusions drawn, warranting further investigation with a larger cohort. Procedures were predominantly conducted by one trained general surgeon; thus, interpersonal dynamics were not thoroughly assessed. Additionally, the reliance on anesthesia team records for operative time may introduce inaccuracies compared to direct surgical monitoring. Pain score assessment on the first postoperative day might be enhanced by considering analgesic requirements alongside patient-reported scores. Furthermore, conducting follow-ups via phone calls around 70 days post-operation raises concerns about potential recall bias in complication evaluation, suggesting physical assessments would be preferable.

Despite these limitations, the study’s pioneering nature provides valuable insights into a novel surgical approach within the regional context. Its direct comparison with conventional options aids comprehension and encourages acceptance among interested surgeons. Meticulous recording and assessment of both intra and postoperative parameters bolster its credibility. The success achieved by a trained general surgeon underscores the importance of training both staff and resident surgical trainees to replicate similar outcomes in the future.

### Electronic supplementary material

Below is the link to the electronic supplementary material.

Supplementary Material 1

## Data Availability

Data is provided within the manuscript or supplementary information files.
