# Supplementary material for: Advancing laparoscopy in resource-limited settings
Source: BMC Surg. 2024 Mar 26;24:98. doi: 10.1186/s12893-024-02387-2 (PMC10964563; doi:10.1186/s12893-024-02387-2)
Supplement: Supplementary file 1 — Supplementary Material 1 [file 12893_2024_2387_MOESM1_ESM.docx]

**Comparison of Procedure Duration Vs Operative Duration**

|  |  | Procedure Duration Range | Procedure Type |
| --- | --- | --- | --- |
| Procedure Duration Range | Correlation Coefficient |  | .289^**^ |
|  | Sig. 2 tailed |  | .001 |
|  | N |  | 119 |
| Procedure Type | Correlation Coefficient | .289^**^ |  |
|  | Sig. (2-tailed) | .001 |  |
|  | N | 119 |  |

Supplementary Table 1

**Comparison of Procedure Type Vs Post Operative Pain**

|  |  | Procedure Duration Range | Procedure Type |
| --- | --- | --- | --- |
| Procedure Type | Correlation Coefficient |  | -.214^*^ |
|  | Sig. 2 tailed |  | .029 |
|  | N |  | 104 |
| Post OP pain group | Correlation Coefficient | .289^**^ |  |
|  | Sig. (2-tailed) | .001 |  |
|  | N | 119 |  |

Supplementary Table 2

**Correlation of Procedure Type Vs Hospital Stay**

|  |  | Procedure Duration Range | Procedure Type |
| --- | --- | --- | --- |
| In-patient Stay | Correlation Coefficient |  | -.862 |
|  | Sig. 2 tailed |  | .000 |
|  | N |  | 119 |
| Procedure type | Correlation Coefficient | -.862 |  |
|  | Sig. (2-tailed) | .000 |  |
|  | N | 119 |  |

Supplementary Table 3
